# Optimization of Canned Talang Queenfish Color Sterilized by Rotary Retort: Storage Stability, Artificial Intelligence—Adaptive Neuro Fuzzy Inference Systems Modeling TBA Based on Color Attributes

**DOI:** 10.1002/fsn3.70768

**Published:** 2025-08-17

**Authors:** Atheer A. A. Al‐Mtury, Asaad R. Al‐Hilphy, Ammar B. Altemimi, Zheng Ruan, Sabah M. Al‐Shatty, Mohammad Ali Hesarinejad, Tarek Gamal Abedelmaksoud

**Affiliations:** ^1^ Department of Food Science College of Agriculture, University of Basrah Basrah Iraq; ^2^ State Key Laboratory of Food Science and Resources Institute of Nutrition, Nanchang University Nanchang China; ^3^ Department of Food Sensory and Cognitive Science Research Institute of Food Science and Technology (RIFST) Mashhad Iran; ^4^ Food Science Department Faculty of Agriculture, Cairo University Giza Egypt

**Keywords:** ANFIS, canned fish, color, optimization process, sterilization

## Abstract

This study aimed to improve the color quality of sterilized Talang Queenfish. It also optimized sterilization conditions using Response Surface Methodology (RSM) with a central composite design. The effect of storage on color attributes was evaluated. Sterilization temperatures tested were 110°C, 115°C, and 121°C, with durations of 10, 20, and 30 min. Two methods were compared: rotary sterilizer (RS) and conventional autoclave (CA). Color parameters measured included L*, a*, b*, Δ*E*, hue angle (*h*), chroma (*C*), whiteness index (WI), yellowness index (YI), and browning index (BI). The Adaptive Neuro‐Fuzzy Inference System (ANFIS) was used to model thiobarbituric acid‐reactive substances (TBA). Optimal conditions were 116°C for 15.54 min. Under these conditions, the RS method improved L*, a*, Δ*E*, *C*, *h*, and WI compared to CA. These values reached 38.77, 31.25, 64.47, 0.16, 45.32, and 39.29, respectively. In contrast, YI and BI decreased by 27.94% and 32.82%. The RS method significantly enhanced color attributes immediately and during storage. ANFIS with a gaussmf membership function accurately predicted TBA. The sixth reduced model achieved the lowest prediction error. These results indicate that color parameters can be used as quality indicators in fish processing.

## Graphical abstract

The SRSIR method significantly improved color attributes under optimal conditions of 116°C for 15.54 min, compared to CA. YI and BI decreased, while color parameters were enhanced immediately and during storage. ANFIS accurately predicted TBA, with the sixth reduced model having the lowest prediction error, indicating color parameters can be used as quality indicators in fish processing.

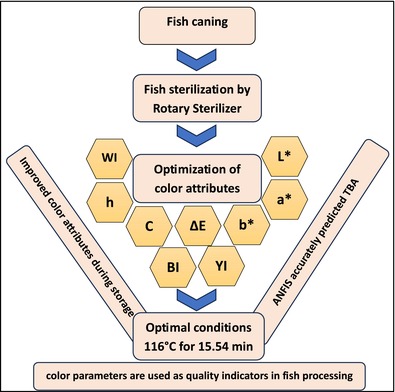

## 1 Introduction

The visual appearance of food, particularly its surface color, serves as a critical indicator of product quality and significantly influences consumer acceptance prior to any sensory evaluation. Among various quality parameters, color is frequently used by consumers as a primary criterion for accepting or rejecting a food product, especially in the absence of other sensory cues (Abdulla et al. 2004; Hatcher et al. 2004; Du and Sun 2004; Pedreschi et al. 2000). Color is widely recognized as one of the most important sensory attributes in both fresh and processed foods. It not only enhances the initial visual appeal but also strongly shapes consumer preferences and perceptions. From a physical standpoint, color perception arises from the interaction of light—defined by its intensity and wavelength—with a material, which is then detected by the human eye (Costa et al. 2011; Sahin and Sumnu 2006). Many researchers have underscored the critical role of color in influencing consumer perceptions of food quality. As a primary visual cue, color is strongly associated with key sensory attributes such as flavor and taste, and it also serves as an indicator of essential quality parameters, including nutritional composition and overall product integrity. In addition, color analysis offers a non‐destructive method for detecting defects and evaluating the effects of thermal processing, especially in canned foods (Spence 2015; Morsy 2016). Food color is commonly assessed using the CIE Lab* color space, introduced in 1976 by the Commission Internationale de l'Éclairage. This system quantifies color through three parameters: L* represents lightness, with higher values indicating lighter shades; a* denotes chromaticity on the red‐green axis, where positive values indicate redness and negative values indicate greenness; and b* reflects chromaticity on the yellow‐blue axis, with positive values corresponding to yellow and negative values to blue. This colorimetric evaluation is typically performed using a colorimeter or digital image analysis systems (Gulrajani 2010; Pathare et al. 2013). The Adaptive Neuro‐Fuzzy Inference System (ANFIS), also referred to as the adaptive‐network‐based fuzzy inference system, is a hybrid modeling approach that integrates fuzzy logic with the learning capabilities of artificial neural networks. ANFIS employs a set of fuzzy if–then rules and corresponding membership functions, which are derived from empirical input–output datasets using a hybrid learning algorithm. This technique has demonstrated superior performance in terms of convergence speed, learning efficiency, and predictive accuracy when compared to conventional modeling methods, making it a valuable tool across various domains, including food science and agriculture (Miao et al. 2023; Nosratabadi et al. 2021). Considering the limited research available on optimizing color attributes in fresh and canned meat and fish products, as well as the need to establish optimal thermal sterilization parameters (i.e., temperature and time), the present study aims to improve the color quality of canned Labeobarbus (Shabout) fish using a rotary retort system. Additionally, the study will compare this method with traditional sterilization techniques and apply ANFIS‐based artificial intelligence modeling to predict Thiobarbituric acid (TBA) values, serving as an indicator of lipid oxidation in the final canned product.

## 2 Materials and Methods

### 2.1 Sample Collection and Canning Procedure

Talang Queenfish ( *Scomberoides commersonianus* ) samples were procured from a local market in Basra Governorate. The samples were rinsed with tap water, thoroughly cleaned, and cut into uniform pieces measuring 4 × 4 cm. These pieces were subsequently blanched at 80°C for 10 min. Following blanching, the fish samples were packed into glass jars with dimensions of 11.7 cm in height and 4.8 cm in diameter. For sterilization, four jars were processed using a rotary retort system, while the remaining four jars were treated using a conventional retort.

### 2.2 Rotary Sterilizer (RS)

A locally fabricated rotary sterilizer, developed by the Department of Food Science at the University of Basrah, was employed in this study. The unit comprises an infrared steam generation module, a superheated steam production system, and a rotating sterilization chamber capable of holding four glass jars. The internal sterilization temperature, along with the cold spot temperatures within the jars, was continuously monitored. Once the target sterilization conditions were achieved (i.e., 115°C for 20 min), the temperature was maintained for the designated period. Following sterilization, the system was deactivated, and the jars were rapidly cooled using tap water. The sterilized samples were subsequently stored at ambient temperature for further analysis.

### 2.3 Conventional Autoclave

The fish samples were canned and subsequently sterilized using a FANEM autoclave. During the sterilization process, both the autoclave chamber temperature and the cold spot temperatures within the cans were continuously monitored. Once the target temperature was achieved, the cans were held at 115°C for 20 min to ensure adequate sterilization. Upon completion of the cycle, the autoclave was deactivated, and the glass cans were rapidly cooled using tap water. The cooled cans were then stored at ambient temperature for subsequent analyses.

### 2.4 Design and Statistical Analysis

Response Surface Methodology (RSM) was used to analyze the data using Central Composite Design (CCD) to describe the variation in experimental data and to predict the effect of the independent variables (temperature and time) on the color components (a*, b*, Δ*E*, C*, *h*, WI, YI, BI). The temperature and time values were determined based on preliminary experiments as follows: 110°C–121°C and 10–20 min, respectively. The color attributes of canned and sterilized fish were improved using the numerical technique. The independent variables were temperature (110°C, 115.5°C, 121°C) and time (10, 15, 20 min), while the dependent variables were a*, b*, Δ*E*, C*, *h*, WI, YI, BI. The Design Expert software, version 13 (Stat‐Ease Inc., USA), was used, and then the desirability functions for the dependent variables were determined. A response surface plot for the dependent variables was also used to select the best interactions for temperature and time. The design included 13 experimental points. Additionally, the coded values were low (−1), medium (0), and high (+1). The desirability function (with values ranging from 0 to 1) improved the multiple responses.

The multiple quadratic regression model Equation (1) was used to predict the dependent variables, Khuri and Cornell (2018):

(1) $$Y=\alpha_{o}+\sum_{i=1}^{k}\alpha_{i}X_{i}+\sum_{i=1}^{k}\alpha_{\mathrm{ii}}X_{i}^{2}+\sum \sum_{i<j=1}^{k-1}\alpha_{\mathrm{ij}}X_{i}X_{j}$$

Where: responses ‘$\alpha_{o}$, $\alpha_{i}$, $\alpha_{\mathrm{ii}},\alpha_{\mathrm{ij}}$: regression constants for the intercept, linear, quadratic, and interaction, respectively. $X_{i}X_{j}$ are independent variables. SPSS version 21 was used to analyze the data using a one‐way ANOVA to determine significance for comparing the expected, experimental, CA, and fresh fish treatments. The LSD test was applied at the 0.05 level.

### 2.5 AI‐ANFIS Modeling

**FIGURE 1:**
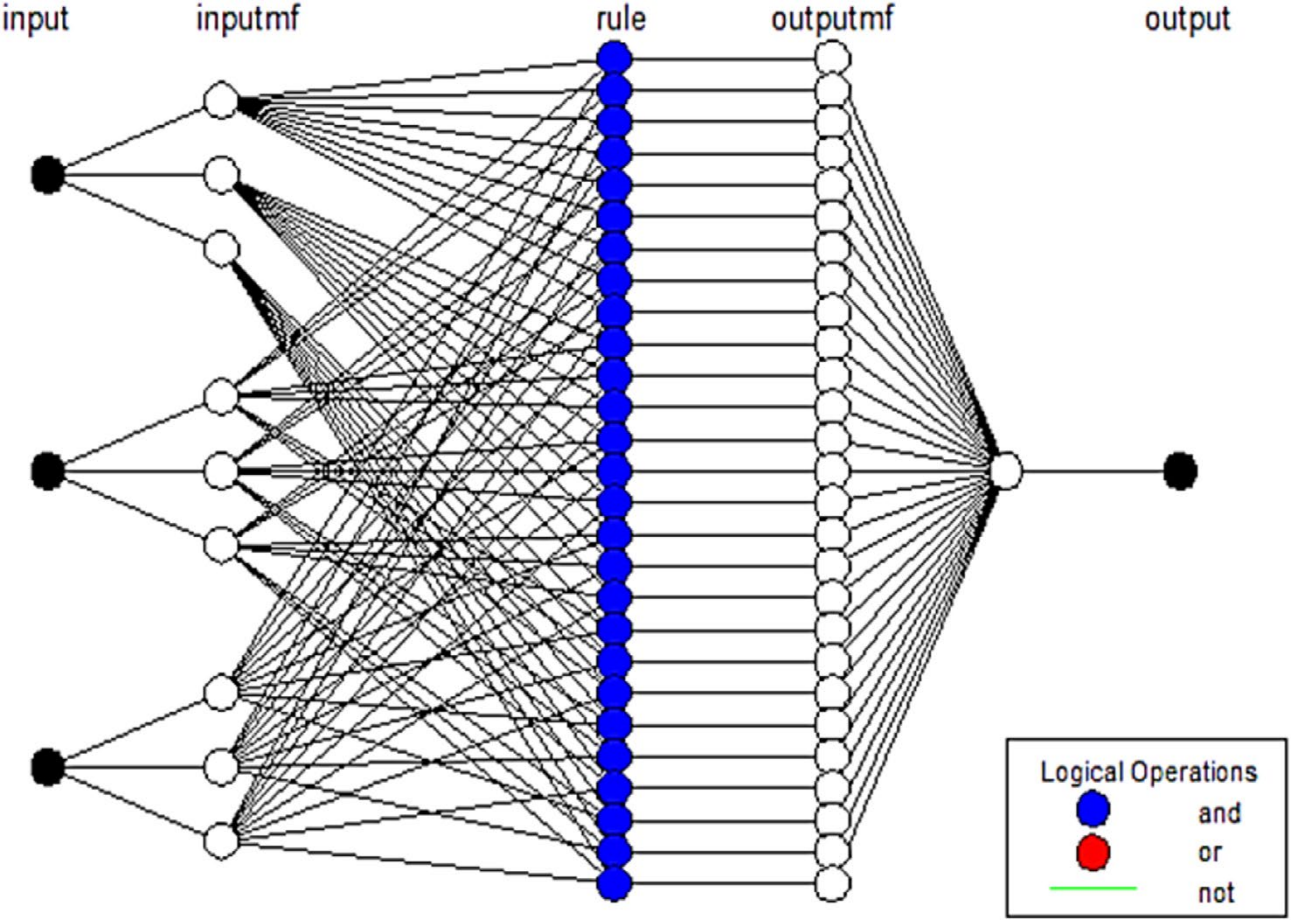
Schematic of the artificial intelligence by neuro‐fuzzy inference system (ANFIS) model methodology used in the research (input: L*, a*, and b*; output: TBA).

ANN and fuzzy logic concepts are combined in compatible deductive neural‐fuzzy systems, which use a set of if‐then fuzzy laws (Abbas et al. 2022; Shihabudheen and Pillai 2018). MATLAB software was used to accomplish neural‐fuzzy modeling in this investigation. To do this, a Sugeno system was used, and function—Gaussian (3 3 3) was chosen as the ideal membership function. Trial and error were also used to earn their membership degree. The fuzzy deductive system was trained and matched using a combinational training technique that included the least square error method and the error backpropagation methodology. The Thiobarbituric acid (TBA) of the sterilized canned fish samples was predicted using this model. The L*, a*, and b* were ANFIS inputs. In the current study, training accounted for 75% of the data, while validation accounted for the remaining 25%. Schematic of the neuro‐fuzzy (ANFIS) model methodology used in the research (Figure 1). The determination coefficient (*R* ^2^) and root mean square error (RMSE) were calculated according to (Equations 2 and 3), respectively.

(2) $$R^{2}=\frac{\sum_{I=1}^{N}{\left(x_{\mathrm{pre}.}-\underline{x_{\mathrm{pre}.}}\right)}^{2}}{\sum_{I=1}^{n}{\left(x_{\exp.}-\underline{x_{\exp.}}\right)}^{2}}$$

(3) $$\text{RMSE}=\sqrt{\frac{\sum_{I=1}^{N}{\left(x_{\exp.}-x_{\mathrm{pre}.}\right)}^{2}}{N}}$$

Where *x*_pre_. is the predicted data, *x*_exp_. is the experimental data, and *N* is the observation number.

### 2.6 Color Attributes

On top of a wooden‐black color box are four light‐emitting diode (LED) lamps (LB13W, Konnice Co., China) and a 720p HD camera (IP67 Endoscope, Mileseey, China). Two lamps are perpendicular to the sample, while the other two are at a 45° angle with the camera lens. The color properties of milk samples were analyzed in triplicate using the image processing technique (Al‐Hilphy et al. 2021). In this context, CIE L*a*b* values were extracted from digital images using the ImageJ software (Version 1.52q, National Institutes of Health, United States) and the acquired digital images.

The L* value (Lightness) indicates the degree of brightness, where 100 represents white and 0 represents black. The a* value reflects the red‐green spectrum, with positive values indicating redness and negative values indicating greenness. The b* value represents the yellow‐blue spectrum, with positive values indicating yellowness and negative values indicating blueness (Arunachalam et al. 2022).

The color change is given by the following (Equation 4).

(4) $$∆E=\sqrt{{\left(L_{o}^{*}-L^{*}\right)}^{2}+{\left(a_{o}^{*}-a^{*}\right)}^{2}+{\left(b_{o}^{*}-b^{*}\right)}^{2}}$$

L* (lightness/darkness), a* (−green/+red), and b* (−blue/+yellow) of pasteurized milk, and $L_{o}^{*},a_{o}^{*},b_{o}^{*}$ of fresh milk.

Chroma (C*) means vividness or saturation of color, and C* is calculated from (Equation 5)

(5) $$C^{*}=\sqrt{{\left(a^{*}\right)}^{2}+{\left(b^{*}\right)}^{2}}$$

The degree of the primary spectral colors, such as red, green, and blue, is referred to as the hue angle. The *h* scales from 0 to 360. The color is red when *h* is 0 or 360, and yellow, green, and blue when *h* is 90, 180, and 290, respectively. *h* is calculated from (Equation 6) as follows:

(6) $$h=\tan^{-1}\left[\frac{a^{*}}{b^{*}}\right]$$

whiteness index (WI) is the whiteness degree and mathematically combines lightness, yellowness, and blueness, as illustrated in (Equation 7):

(7) $$\mathrm{WI}=100-\sqrt{{\left(100-L^{*}\right)}^{2}+{\left(a^{*}\right)}^{2}+{\left(b^{*}\right)}^{2}}$$

The measurement associated with the browning index is called the yellowness index (YI), and it is given in (Equation 8):

(8) $$\mathrm{YI}=142.86\left[\frac{b^{*}}{L^{*}}\right]$$

The browning index (BI) is a measure of the purity of a brown hue that describes the browning of sugar‐containing foods and is derived from Equation (9) [14]:

(9) $$\mathrm{BI}=\frac{100\left(X-031\right)}{0.172}$$

(10) $$X=\frac{a^{*}+1.75L^{*}}{6.645L^{*}+a^{*}-3.012b^{*}}$$

The color index in food processing is vital for ensuring product quality and consumer satisfaction, employing standardized measurement techniques to achieve consistent and appealing food color.

### 2.7 Thiobarbituric Acid (TBA) Assay

The procedure was conducted according to the method outlined by Saad et al. (2021). In brief, 20 g of the sample was combined with 100 mL of 7.5% trichloroacetic acid solution and thoroughly homogenized using a magnetic stirrer for 2 min. The homogenate was then filtered through filter paper. Subsequently, 5 mL of the resulting filtrate was mixed with 5 mL of 0.02 M TBA reagent in a test tube. The test tube was placed in a boiling water bath for 40 min. Following incubation, the absorbance of the developed pink color was measured at 538 nm using a spectrophotometer. TBA values were calculated using the following equation:

$$\mathrm{TBA}=\frac{0.016+2.782}{10}-X\;\mathrm{mg}/100g$$

*X* = absorbance of the sample.

## 3 Results and Discussion

### 3.1 Color Parameters of Canned Talang Queenfish

#### 3.1.1 *L* Value (Lightness)*

Table 1 presents the central composite design matrix illustrating the effect of sterilization temperature and time on the color components of canned Talang Queenfish. The results showed that the highest lightness (L*) value was 56.2 at 121°C for 15 min, while the lowest value was 52.3 at 110°C for 20 min. This reduction in L* is attributed to the longer exposure time at the sterilization temperature.

**TABLE 1:** Central composite design matrix for the impact of temperature and time of sterilization on the color components of sterilized fish and color indexes.

Run	*T* (°C)	*t* (min)	L*	a*	b*	Δ*E*	C*	*h* (°)	WI	YI	BI
1	115.5	15	54.4	1.6	18.4	29.21	18.46	4.97	50.80	48.32	41.97
2	110	15	53.2	1.4	17.6	28.54	17.65	4.55	49.98	47.26	40.68
3	115.5	10	54.3	1.5	18.3	29.19	18.36	4.68	50.74	48.14	41.66
4	121	20	55.4	1.5	18.6	30.05	18.66	4.61	51.65	47.96	41.44
5	121	10	55.3	1.4	18.4	30.06	18.45	4.35	51.64	47.53	40.87
6	115.5	15	54.5	1.6	18.4	29.30	18.46	4.97	50.89	48.23	41.88
7	121	15	56.2	1.6	18.2	30.87	18.27	5.02	52.54	46.26	39.85
8	115.5	15	54.3	1.4	18.4	29.18	18.45	4.35	50.71	48.40	41.78
9	110	10	52.4	1.5	17.7	27.79	17.76	4.84	49.19	48.25	41.85
10	115.5	15	55.8	1.7	18.2	30.49	18.27	5.33	52.16	46.59	40.33
11	115.5	15	54.5	1.6	17.5	29.64	17.57	5.22	51.22	45.87	39.53
12	110	20	52.3	1.4	18.3	27.49	18.35	4.37	48.89	49.98	43.46
13	115.5	20	54.6	1.5	18.5	29.38	18.56	4.63	50.95	48.40	41.91

Abbreviations: a*, redness/blueness; b*, yellowness/greenness; BI, browning index; C*, Chroma; *h*, hue angle; L*, lightness; *T*, temperature; *t*, time; WI, whiteness index; YI, yellowness index; Δ*E*, total color differences.

Statistical analysis results in Table S1 revealed that the quadratic model (QM) and temperature had a significant effect (*p* < 0.05) on the L* value. In contrast, the effects of the remaining factors were not statistically significant (*p* > 0.05). The non‐significant lack of fit (LOF) value indicates a good agreement between the actual and predicted values. The statistical indicators supported the applicability of the QM for predicting L* values, with *R* ^2^ = 0.892, predicted *R* ^2^ = 0.749, and a coefficient of variation (C.V.) of 0.936%. The QM coefficients are detailed in Table S1. Table 2 shows the regression coefficients and *p*‐values for the L* prediction model.

**TABLE 2:** Regression coefficients of mathematical models.

	Intercept	*T*	*t*	Tt	*T* ^2^	*t* ^2^
*L*	−106.3120	2.4573	0.4475	0.0018	−0.00957	−0.02158
*p*‐values		0.0002	0.8170	0.8500	0.3764	0.1219
a*	−25.45689	0.47910	−0.131379	0.00181	−0.00216	−0.00262
*p*‐values		0.4142	1.0000	0.3231	0.2851	0.2851
b*	−87.49655	1.76196	0.11402	−0.00363	−0.00718	0.01131
*p*‐values		0.0670	0.2178	0.5283	0.2701	0.1631
Delta *E*	−55.83499	1.21612	0.36954	0.00258	−0.00449	−0.02239
*p*‐values		0.0004	0.9159	0.7659	0.6375	0.0822
C*	−89.34178	1.79610	0.10377	−0.00348	−0.00733	0.011046
*p*‐values		0.0639	0.2161	0.5427	0.2574	0.1689
*h*	−53.309024	1.06099	−0.44247	0.006628	−0.00499	−0.01106
*p*‐values		0.7867	0.7482	0.2895	0.4559	0.1918
WI	23.719239	0.235511	−0.002887			
*p*‐values		0.0003	0.9523			
YI	−81.165186	2.330119	−0.011856	−0.011829	−0.009810	0.048628
*p*‐values		0.1515	0.3327	0.5152	0.6195	0.0709
BI	−118.839252	2.898374	−0.196804	−0.009498	−0.012432	0.045824
*p*‐values		0.1326	0.3165	0.5871	0.5179	0.0768

**FIGURE 2:**
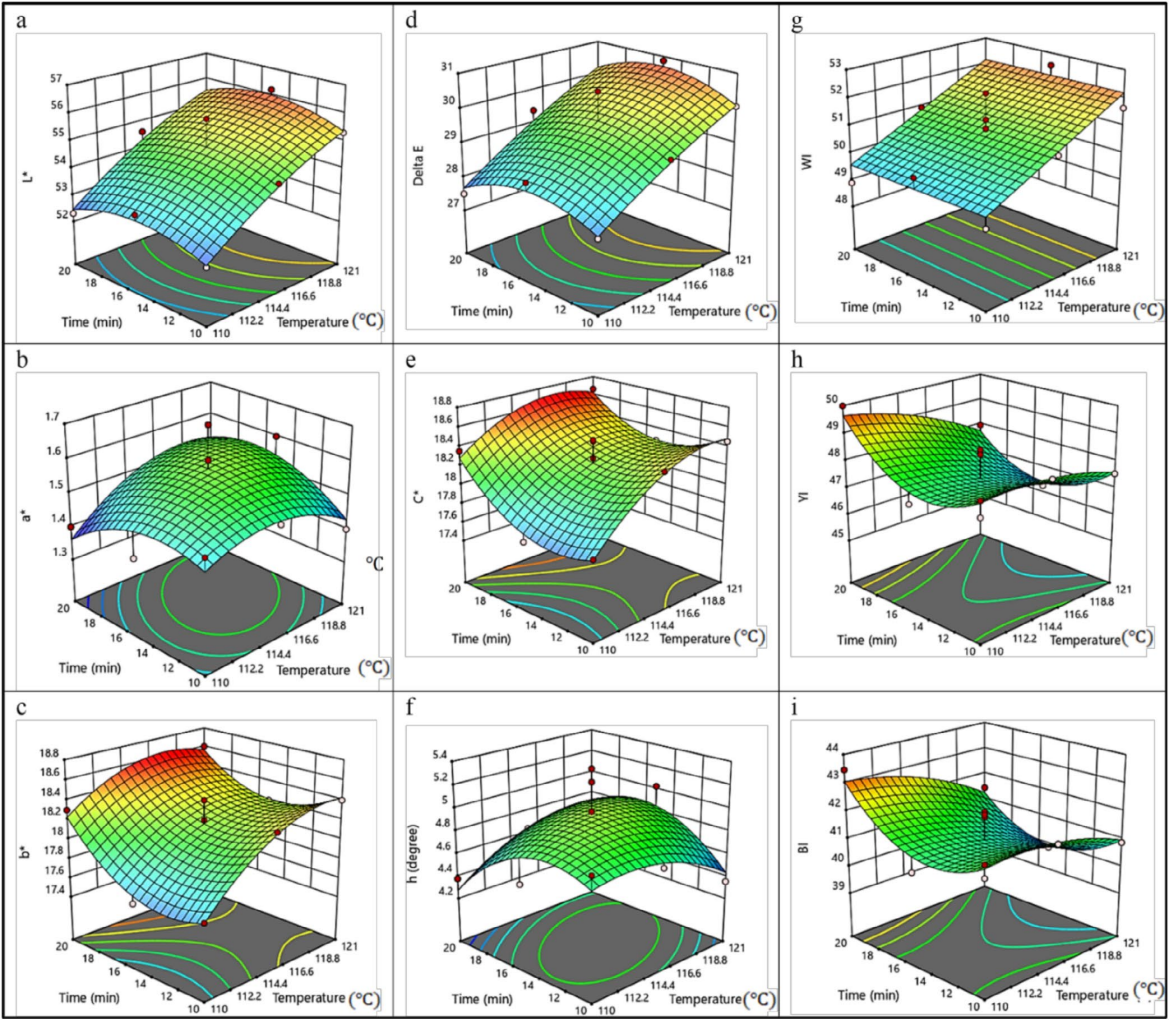
Response surface plot of (a) Lightness (L*), (b) Redness (a*), (c) Yellowness (b*), (d) Total color difference (Δ*E*), (e) Chroma (C*); (f) Hue angle (*h*); (g) Whiteness index (WI); (h) Yellowness index (YI); and (i) Browning index (BI) as a function of process time and temperature.

The 3D surface response plot in Figure 2a demonstrates the interaction effect of the independent variables on L*. The values increased from 52.45 at 110°C and 10 min to higher values at 121°C and 20 min. The findings indicate that temperature had a more pronounced effect than time. No significant differences were observed between 10 and 20 min at 110°C. The findings are consistent with those of Martinez et al. (2024), who reported L* values ranging from 55.54 to 60.39 for canned gilt‐head bream ( *Sparus aurata* ).

#### 3.1.2 *a* Value (Redness)*

According to Table 1, the highest redness value (a*) was 1.7 at 115.5°C for 15 min, while the lowest value of 1.4 was recorded in experiments 2, 5, 8, and 12. These variations are attributed to changes in temperature and sterilization time. There was no significant effect of the quadratic model or any of the factors on a* (*p* > 0.05). Moreover, the statistical indicators were weak, with *R* ^2^ = 0.470, indicating that a* values could not be reliably predicted using the regression model (Table S2). Table 2 provides the regression coefficients and *p*‐values for the a* prediction model. As shown in Figure 2b, the interaction between temperature and sterilization time revealed that a* values were 1.36 at 110°C for 20 min, increased to 1.52 at 121°C for the same duration, and slightly decreased to 1.42 at 121°C. At 116.5°C and 20 min, a* increased to 1.51. These results are consistent with those of Martinez et al. (2024), who reported a* values ranging from 1.91 to 2.18 for canned golden sea bream ( *Sparus aurata* ).

#### 3.1.3 *b* Value*

The b* values ranged between 17.5 at a sterilization temperature of 115.5°C for 15 min and 18.6 at 121°C for 20 min, as shown in Table 1. Results from the ANOVA table (Table S3) indicated that temperature and sterilization time had no significant effect (*p* > 0.05) on the b* value, with low statistical indicators observed (*R* ^2^ = 0.583 and Predicted *R* ^2^ = 0.286). Table 2 presents the regression coefficients and *p*‐values for the prediction model of the b* value. Regarding the interaction between the independent variables (Figure 2c), the b* value increased non‐significantly (*p* > 0.05) with the increase in both temperature and sterilization time. For instance, when the temperature increased from 110°C to 121°C, the b* value changed from 17.79 to 17.70, respectively, at a sterilization time of 10 min. Similarly, when the sterilization time increased from 10 to 20 min, the b* value increased from 17.70 to 18.22 at a temperature of 110°C. The results indicated that the increase in b* values was minimal. These findings are consistent with those reported by Villamarín et al. (2023), who studied the yellowness index (b*) of canned horse mackerel, where b* values ranged from 12.81 to 16.47.

#### 3.1.4 Δ*E* Values

The results in Table 1 show that the Δ*E* values ranged between 27.50 at a temperature of 110°C and a sterilization time of 20 min and 30.87 at 121°C and a sterilization time of 15 min. This variation is attributed to changes in temperature, where Δ*E* increased with rising temperature. The ANOVA results in Table S4 indicated that both the quadratic model (QM) and temperature had a significant effect (*p* < 0.05) on Δ*E*. In contrast, the remaining factors and lack of fit (LoF) showed no significant effects (*p* > 0.05). The statistical indicators were *R* ^2^ = 0.869, Adjusted *R* ^2^ = 0.775, and Predicted *R* ^2^ = 0.666. Table 2 presents the regression coefficients and *p*‐values for the predictive model of Δ*E*.

Δ*E* values can be predicted using the QM model, with its coefficients provided in Table S10. Regarding the interaction between independent factors, the 3D plot (Figure 2d) showed that Δ*E* increased with temperature, while its behavior varied with sterilization time. For example, Δ*E* was 27.66 at 110°C and 20 min, increasing to 30.19 at 121°C at the same time. A slight decrease in Δ*E* was observed when sterilization time increased from 10 to 20 min. The results suggest that temperature plays a more crucial role than sterilization time in affecting Δ*E*. These findings differ from those reported by Gómez‐Limia et al. (2022), who studied the effect of filling media on the color of canned European eel, where Δ*E* values were 10.69, 12.31, and 10.70, respectively.

#### 3.1.5 *C* Value

As shown in Table 1, the lowest Chroma (*C*) value was 17.57 at 115.5°C and 15 min, while the highest value was 18.45 at 121°C and 10 min. According to the ANOVA results in Table S5, the quadratic model and all other factors showed no significant effects, and the statistical indicators were low. Therefore, Chroma *C* cannot be predicted using the QM model. Regression coefficients and *p*‐values are presented in Table 2. Figure 2e illustrates that *C* increased with both temperature and sterilization time. The lowest value, 17.76, was recorded at 110°C and 10 min, while the highest value, 18.62, was recorded at 121°C and 20 min. These results agree with those of Gómez‐Limia et al. (2022), who found Chroma *C* values of 19.69, 20.34, and 19.75 when studying the effect of filling media on the color indices of canned European eel.

#### 3.1.6 *h* Value

The hue angle (*h*) ranged from 4.35 (trial 5) to 5.34 (trial 10) as shown in Table 1. This variation is attributed to changes in a* and b* values, as previously explained.

No significant differences (*p* > 0.05) were found for the quadratic model or any factors affecting *h*, as indicated in Table S6. Due to low statistical indicators, the QM model cannot reliably predict *h*. Table 2 displays regression coefficients and *p*‐values for the predictive model of *h*. Figure 2f shows that h varied with temperature and sterilization time. Increasing temperature from 110°C to 121°C raised *h* from 4.27 to 4.71 at 20 min, while at 10 min, *h* decreased from 4.72 to 4.43. These findings differ from those of Galhoum (2020), who reported *h* values of 74.84 and 76.90 for local and imported canned tuna, respectively.

#### 3.1.7 Whiteness Index (WI)

Table 1 shows that WI values ranged between 48.8 (trial 12) and 52.72 (trial 8). These differences are attributed to changes in L*, a*, and b* values due to temperature and sterilization time. ANOVA results in Table S7 showed that the linear model (LM) and temperature had a significant effect (*p* < 0.05) on WI, while sterilization time and LoF did not (*p* > 0.05). The *R* ^2^, Adjusted *R* ^2^, and Predicted *R* ^2^ values were 0.752, 0.702, and 0.545, respectively, indicating the feasibility of predicting WI. Coefficients of the linear model are shown in (Table 2). Regarding interaction effects, (Figure 2g) showed that increasing temperature led to higher WI at all sterilization times. For instance, at 10 min, WI increased from 49.59 to 52.18 as temperature rose from 110°C to 121°C. These results differ from those of Galhoum (2020), who reported WI values of 3.21 and 2.33 for local and imported canned tuna, respectively.

#### 3.1.8 Yellowness Index (YI)

Table 1 indicates that the highest YI value was 49.99 at 110°C and 20 min, while the lowest was 45.87 at 115.5°C and 15 min. ANOVA results in Table S8 revealed no significant effects (*p* > 0.05) from the quadratic model or any factors on YI, so the model cannot be used for prediction. Table 2 provides regression coefficients and *p*‐values for the predictive model of YI. The interaction between the two independent variables showed a slight decrease in YI with increased temperature and reduced sterilization time. For example, at 10 min, YI decreased from 48.17 to 47.58 as temperature increased from 110°C to 121°C. At 20 min, YI decreased from 49.63 to 47.73. The magnitude of reduction was greater at 110°C compared to 121°C. For example, increasing the sterilization time from 10 to 20 min reduced YI from 49.63 to 48.45 at 110°C. Differences decreased significantly at 121°C.

#### 3.1.9 Browning Index (BI)

According to (Table 1), the lowest and highest BI values were 39.54 and 41.98, both observed at 115.5°C and 15 min. Table S9 showed that BI was not significantly affected (*p* > 0.05) by the quadratic model or any factors. Table 2 lists regression coefficients and *p*‐values for BI prediction. As illustrated in (Figure 2i), increasing temperature and reducing sterilization time led to a decrease in BI. For instance, increasing temperature from 110°C to 121°C resulted in a BI drop from 40.96 to 41.71 at 10 min. Meanwhile, reducing sterilization time from 20 to 10 min lowered BI from 43.04 to 41.71 at 110°C.

### 3.2 Optimization Results

As shown in Table 3, the L* value of the retort sterilized (RS) fish was significantly higher (*p* < 0.05) than that of conventionally sterilized (CA) and fresh fish. This can be attributed to the movement of the brine surrounding the canned fish and the thermal effect, which lightened the color. The a* value was significantly higher (*p* < 0.05) in RS compared to CA; however, both RS and CA showed significantly lower (*p* < 0.05) a* values than fresh fish due to the heat‐induced chemical reactions in fish tissues. No significant differences (*p* > 0.05) were found between RS and CA in b* and Chroma (*C*), though both differed significantly from fresh fish. Δ*E* was higher in RS than in CA. As for h, it was significantly higher (*p* < 0.05) in RS compared to CA, but both were significantly lower than in fresh fish. Significant differences (*p* < 0.05) in WI were observed between RS and CA, with WI being highest in RS, followed by CA and fresh fish, attributed to the increased L* value. The browning index (BI) was reduced by 34.52% and 56.76% in RS compared to CA and fresh fish, respectively.

**TABLE 3:** Optimization results.

*T* (°C)	*T* (min)	Color components	Predicted	Experimental	Conventional autoclave	Fresh fish
116	15.54	L*	54.92^a^	54.40^a^	39.20^c^	42.40^b^
		a*	1.58^b^	1.60^b^	1.10^a^	16.60^c^
		b*	18.21^a^	18.40^a^	18.41^a^	21.20^b^
		Delta *E*	29.75^a^	29.21^a^	17.76^b^	—
		C*	18.27^a^	18.46^a^	18.43^a^	26.93^b^
		*h*	4.96^b^	4.97^b^	3.42^a^	38.08^c^
		WI	50.99^a^	50.80^a^	36.47^b^	36.42^b^
		YI	47.36^a^	48.32^a^	67.06^b^	71.43^c^
		BI	40.97^a^	41.97^a^	62.48^b^	94.59^c^
		Desirability	0.857			

Abbreviations: a*, redness/blueness; b*, yellowness/greenness; BI, browning index; C*, Chroma; *h*, hue angle; L*, lightness; *T*, temperature; *t*, time; WI, whiteness index; YI, yellowness index; Δ*E*, total color differences. Different lowercase letters within a row indicate statistically significant differences (*p* ≤ 0.05).

### 3.3 Color Attributes Changes During Storage Periods

#### 3.3.1 *L* Value*

**FIGURE 3:**
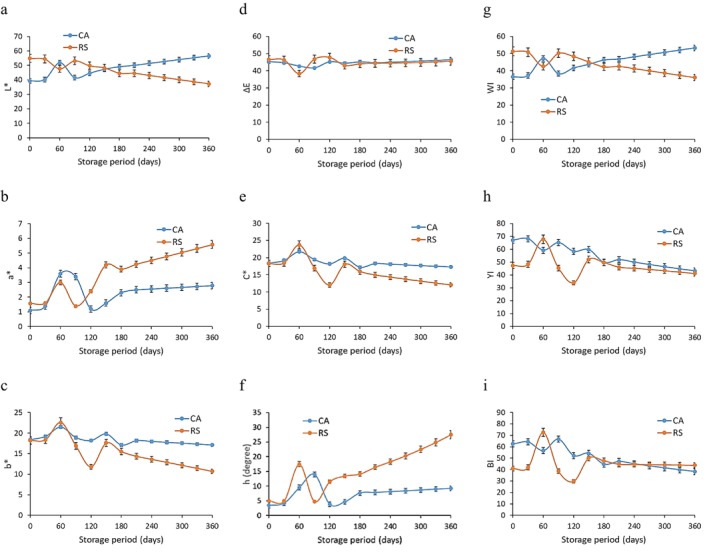
Changing color attributes of Talang Queenfish sterilized by RS and CA during storage.

Figure 3a illustrates the L* (lightness) values of canned and sterilized Talang Queenfish using RS (retort sterilization) and CA (conventional autoclaving) methods throughout the storage period (0–360 days). A higher L* value (57.3) was recorded for Talang Queenfish sterilized by RS at Day 0, which subsequently decreased to 39.8 by the end of storage. This decline may be attributed to the formation of micro‐cracks on the surface during storage due to dehydration caused by the sterilization process, leading to lower L* values. Additionally, oxidative reactions resulting from heat exposure can affect surface color and reduce L*. Moisture loss due to sterilization may also increase surface roughness, contributing to the reduction in L* values. For Talang Queenfish sterilized by CA, Figure 3a shows a lower initial L* value of 40.2, which increased to 58.9 at the end of the storage period (360 days). These findings are consistent with those of Martinez et al. (2024), who reported L* values ranging from 55.54 to 60.39 in canned gilt‐head bream. However, the results differ from those of Cruz et al. (2022), who found L* values of 35.9, 31.0, and 35.2 in canned sardines stored in brine, vegetable oil, and tomato sauce, respectively, at 4°C for 1 week.

#### 3.3.2 *a* Value*

Figure 3b shows the a* (redness) values of canned Talang Queenfish sterilized by RS and CA during storage. An increase in a* was observed in the RS group, rising from 1.8 at Day 0 to 5.7 at Day 360. Similarly, in the CA group (Figure 3b), a* increased from 1.3 to 2.9 during the same period. This increase could be due to oxidative reactions caused by high temperatures, leading to color changes and increased a* values. The breakdown of natural pigments such as hemoglobin may also contribute to the rise in a*. These results are comparable to those of Martinez et al. (2024), who reported a* values ranging from 1.91 to 2.18. However, they differ from Cruz et al. (2022), who found a* values of −0.8, −0.8, and 17.5 for canned sardines stored in three different preservation media.

#### 3.3.3 *b* Value*

Figure 3c illustrates the b* (yellowness) values of Talang Queenfish sterilized using RS and CA. For the RS method, b* started at 23.6 on day 0 and decreased to 11.4 by day 360. For CA, b* decreased from 21.2 to 19.4 over the same period (Figure 3c). The reduction in b* may be linked to the high moisture generated during steam sterilization, which reduces Maillard reactions and helps maintain higher b* values. To optimize b* values during storage, precise control of temperature and exposure time is necessary to minimize Maillard reactions. These results are similar to those of Villamarín et al. (2023), who reported b* values between 16.47 and 18.81 for canned horse mackerel. However, they differ from Cruz et al. (2022), who observed b* values of 4.1, 1.9, and 20.4 in canned sardines stored for 1 week.

#### 3.3.4 Δ*E* Value

Figure 3d presents the Δ*E* (total color difference) values of Talang Queenfish sterilized using RS and CA. For RS, Δ*E* decreased from 20.1 on Day 0 to 16.3 on Day 360. In contrast, the CA group showed an increase from 17.2 to 21.2 over the same period. This can be attributed to the reduced Maillard reactions and enhanced chemical stability offered by steam sterilization, which provides better protection against color changes during storage. These findings contrast with Gómez‐Limia et al. (2022), who reported Δ*E* values of 10.69, 12.31, and 10.70 for canned European eel. They also differ from Cruz et al. (2022), who reported Δ*E* values of 1.3, 1.1, and 2.3 for sardines preserved in three different media.

#### 3.3.5 Chroma (*C*) Value

Figure 3e shows the chroma (*C*) values for Talang Queenfish sterilized by RS and CA. For the RS method, chroma decreased from 18.7 on day 0 to 13.4 on day 360. In the CA group, the value slightly declined from 19.8 to 18.7. These findings align with Gómez‐Limia et al. (2022), who recorded *C* values of 19.69, 20.34, and 19.75 for canned European eel.

#### 3.3.6 Hue Angle (*h*°) Value

Figure 3f displays the hue angle (*h*°) values of Talang Queenfish sterilized by RS and CA over storage. In the RS group, *h*° increased from 4.97 on Day 0 to 29.8 at Day 360. For CA, *h*° rose from 4.3 to 9.8 over the same period. This increase can be attributed to Maillard reactions induced by high heat, leading to color shifts toward yellow or brown. These results differ from those of Galhoum (2020), who found *h*° values of 74.84 and 76.90 for local and imported canned tuna, respectively.

#### 3.3.7 Whiteness Index (WI)

Figure 3g presents the WI values of Talang Queenfish sterilized using RS and CA during storage (0–360 days). In the RS group, WI decreased from 52.6 to 39.4, while in the CA group, it increased from 38.6 to 55.2. The reduction in WI may be due to Maillard reactions induced by high heat, leading to the formation of yellow or brown melanoidin compounds that reduce WI. These findings differ from Galhoum (2020), who reported WI values of 3.21 and 2.33 for local and imported canned tuna, respectively.

#### 3.3.8 Yellowness Index (YI)

Figure 3h illustrates the YI values of Talang Queenfish sterilized using RS and CA during storage (0–360 days). For RS, YI decreased from 49.8 on Day 0 to 40.4 on Day 360. In contrast, the CA group showed a decline from 68.7 to 42.8. The decrease in YI may be due to the reduced oxidation resulting from steam sterilization, which provides better protection and lowers YI values over time.

#### 3.3.9 Browning Index (BI)

Figure 3i shows the BI values of Talang Queenfish sterilized by RS and CA. For RS, BI increased from 41.4 at Day 0 to 48.2 at Day 360. In contrast, BI in the CA group decreased from 63.5 to 40.8. The increase in BI may result from Maillard reactions caused by high heat, leading to the formation of brown melanoidin compounds that contribute to higher BI values.

### 3.4 Modeling Thiobarbituric Acid (TBA) Based on the Color Attributes

A 100‐experiment was executed to measure TBA based on the color attributes (L*, a*, and b*) as illustrated in Table 4. For predicting TBA, the reduced sixth model was used as depicted in (Equation 11). The statistical indicators were C.V. (%), Adjusted *R* ^2^, Predicted *R* ^2^, and Adeq Precision, which are 0.4522, 0.9996, 0.9925, and 449.0511, respectively (Table S10). In addition, the model, L*, a*, b*, and all the interactions were significant (Table S10).

(11) $$\begin{array}{cc} \mathrm{TBA}\;=\; & 1553.907-83.553L-1428.1105a+25.135b\\ & +39.986L\;a+1.083L^{2}+557.833a^{2}-0.054L^{2}\;a\;b\\ & -0.00437L^{3}\;a+0.00028L^{3}\;b-13.794L\;a^{3}\\ & -0.02817a\;b^{3}+0.00327L^{2}\;a\;b^{2}+0.00049L\;a^{2}\;b^{2}\\ & +0.0044L^{3}\;a^{2}+7.1665\times 10^{-05}L^{3}\;b^{2}-0.00014L^{2}\;b^{3}\\ & +0.0898a^{3}*b^{2}-2.288610^{-05}L^{4}\;b+6.145L\;a^{4}\\ & -25.1124a^{5}+0.00216L^{3}\;a^{3}-0.00045L^{3}\;a\;b^{2}\\ & +0.00128L^{2}*a\;b^{3}+0.001155L\;a^{3}\;b^{2}\\ & -0.00034L\;a^{2}\;b^{3}-7.90710^{-05}L^{4}\;a^{2}\\ & +7.797910^{-05}L^{4}\;a\;b-3.790810^{-07}L^{4}\;b^{2}\\ & -0.065019L^{2}\;a^{4}+2.28110^{-06}L^{2}*b^{4}\\ & -0.001979L\;a\;b^{4}-4.392610^{-06}L^{5}\;a\\ & +1.540810^{-07}L^{5}\;b-0.22278a^{5}\;b+0.001224a\;b^{5}\\ & -1.8809\times 10^{-09}L^{6}+2.3273a^{6} \end{array}$$

**TABLE 4:** The effect of color attributes on the experimental TBA.

Exp. No.	L*	a*	b*	TBA	Exp. No.	L*	a*	b*	TBA	Exp. No.	L*	a*	b*	TBA	Exp. No.	L*	a*	b*	TBA
1	54.12	1.69	18.49	0.28	26	54.36	1.51	17.61	0.23	51	53.90	1.38	18.44	0.24	76	51.33	1.65	17.19	0.15
2	53.94	1.71	18.33	0.29	27	54.93	1.45	17.63	0.19	52	53.51	1.37	18.55	0.25	77	52.48	1.86	17.30	0.28
3	54.46	1.67	18.42	0.27	28	54.99	1.39	17.64	0.18	53	54.81	1.29	18.82	0.20	78	51.81	1.60	17.37	0.19
4	53.93	1.68	18.44	0.28	29	54.46	1.52	17.68	0.23	54	55.02	1.50	18.51	0.20	79	52.21	1.91	17.61	0.28
5	54.24	1.52	18.35	0.23	30	54.41	1.52	17.49	0.23	55	54.91	1.39	18.52	0.19	80	50.85	1.89	17.51	0.12
6	53.43	1.72	18.37	0.28	31	56.79	1.76	18.55	0.23	56	52.88	1.37	18.71	0.26	81	53.96	1.69	17.69	0.28
7	52.99	1.58	18.51	0.24	32	56.21	1.71	18.38	0.23	57	52.79	1.41	18.49	0.25	82	53.73	1.48	17.72	0.24
8	53.27	1.51	18.40	0.24	33	55.53	1.59	18.30	0.20	58	53.15	1.30	18.65	0.28	83	54.54	1.61	17.53	0.25
9	53.47	1.42	18.51	0.25	34	56.15	1.64	18.17	0.19	59	53.65	1.47	18.56	0.24	84	53.89	1.84	17.46	0.35
10	53.20	1.75	18.51	0.28	35	55.95	1.79	18.39	0.30	60	52.83	1.47	18.70	0.24	85	54.79	1.61	17.42	0.24
11	54.13	1.43	18.30	0.23	36	52.35	1.67	18.20	0.23	61	53.57	1.41	17.60	0.24	86	52.82	1.58	17.62	0.24
12	54.15	1.42	18.53	0.23	37	52.37	1.71	18.39	0.23	62	53.93	1.50	17.56	0.24	87	54.11	1.60	17.44	0.25
13	54.07	1.33	18.53	0.24	38	51.93	1.74	18.25	0.21	63	53.94	1.64	17.72	0.26	88	52.25	1.50	17.55	0.22
14	53.97	1.34	18.16	0.24	39	52.40	1.72	18.58	0.24	64	53.76	1.56	17.53	0.25	89	54.61	1.44	17.74	0.21
15	54.17	1.39	18.42	0.23	40	51.77	1.71	18.26	0.19	65	54.24	1.44	17.51	0.22	90	53.34	1.56	17.52	0.25
16	55.55	1.45	17.50	0.15	41	56.02	1.59	18.28	0.17	66	55.95	1.49	17.94	0.13	91	56.18	1.66	18.21	0.20
17	56.36	1.45	17.44	0.08	42	56.15	1.55	18.34	0.14	67	56.19	1.52	17.42	0.12	92	54.39	1.31	18.09	0.22
18	55.41	1.48	17.76	0.17	43	55.82	1.60	18.45	0.19	68	56.41	1.54	17.78	0.11	93	54.64	1.30	18.28	0.21
19	55.55	1.41	17.72	0.14	44	55.83	1.41	18.30	0.12	69	55.99	1.54	17.56	0.14	94	55.62	1.26	18.37	0.12
20	55.45	1.34	17.58	0.14	45	56.02	1.70	18.31	0.23	70	55.63	1.39	17.85	0.13	95	54.90	1.24	18.32	0.19
21	55.51	1.49	17.65	0.16	46	54.74	1.66	18.53	0.26	71	52.26	1.69	17.63	0.22	96	55.74	1.38	18.32	0.12
22	55.19	1.50	17.64	0.19	47	54.76	1.61	18.33	0.24	72	52.80	1.68	17.73	0.25	97	55.21	1.30	18.10	0.16
23	55.63	1.36	17.87	0.13	48	54.19	1.38	18.52	0.23	73	51.80	1.57	17.83	0.19	98	56.11	1.69	17.80	0.22
24	55.13	1.57	17.51	0.21	49	55.20	1.57	18.71	0.21	74	51.64	1.82	17.63	0.19	99	55.01	1.55	18.02	0.21
25	55.24	1.44	17.71	0.17	50	54.20	1.64	18.62	0.26	75	52.59	1.84	17.65	0.28	100	54.28	1.55	18.28	0.24

Abbreviations: a*, redness‐blueness; b*, yellowness‐greenness; Exp. No., experiments numbers; L*, Lightness; TBA, Thiobarbituric acid.

**FIGURE 4:**
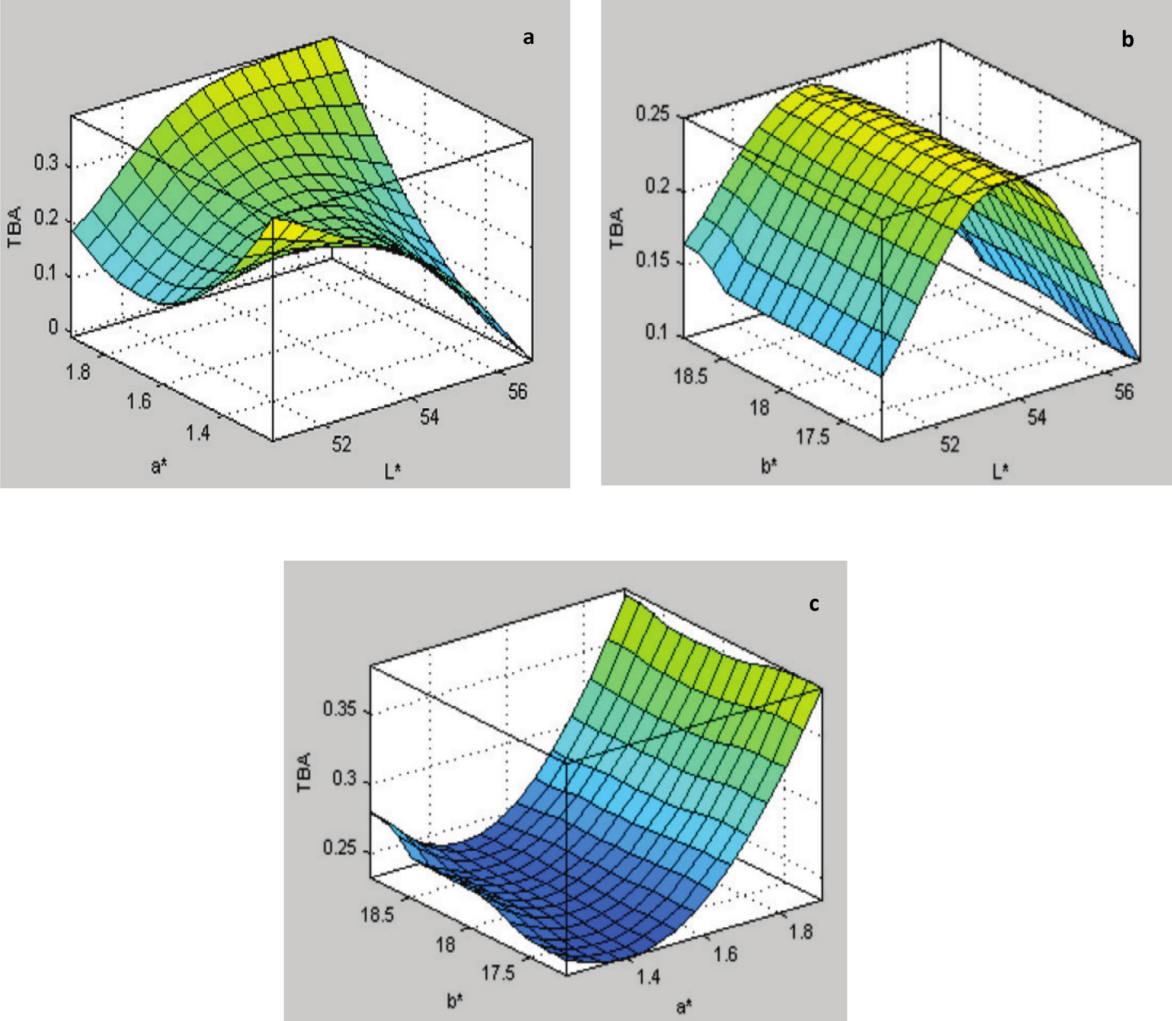
Predicted TBA by artificial intelligence‐ANFIS based on the color attributes.

3D Figure 4 explained the predicted TBA by artificial intelligence‐ANFIS based on the color attributes. Figure 4a illustrates the effect of L*, a* on TBA. So, when L* is 79.52 and a* is 1.41, TBA reached 0.250 mg malondialdehyde/kg fish. In addition, when the value of L* was increased to 56.19 and a* to 1.69, the TBA value reached 0.218 mg malondialdehyde/kg fish. The increase in L* and a* led to a decrease in TBA by 12.8%. Moreover, when the value of a* decreased to 1.52 while the value of L* remained at 56.19, the value of TBA decreased by 46%.

As shown in the results in Figure 4b, which illustrated the effect of L* and b* on TBA, the maximum value of TBA was 0.222 mg malondialdehyde/kg of fish at L* of 54.24 and b* of 17.51. The lowest value of TBA was 0.114 mg malondialdehyde/kg of fish at L* of 56.41 and b* of 17.75. The TBA value increases slightly with an increase in b* while the L* value remains constant, but the TBA value was variable with L*.

Figure 4c illustrates the relationship between a*, b*, and TBA. The results showed that an increase in the value of a* led to an increase in the value of TBA. For example, when the value of a* increased from 1.84 to 1.38, the value of TBA increased from 0.229 to 0.284 mg malondialdehyde/kg fish at b* of 17.76. The increase in a* indicates an increase in redness, which suggests a darker color and signifies increased oxidation. The increase in TBA value was slight with the increase in b*. The effect of the value of a* on the TBA value was greater than the value of b*.

## 4 Conclusions

The response surface methodology (RSM) employing a central composite design (CCD) was utilized to optimize the color parameters of canned and sterilized fish processed using a rotary sterilizer (RS) and a conventional autoclave (CA). The RS method significantly influenced various color attributes, including L*, a*, b*, Δ*E*, chroma (*C*), hue angle (*h*), yellowness index (YI), and browning index (BI). Optimal sterilization conditions using the RS were identified as 116°C for 15.54 min. The L* and Δ*E* values were accurately predicted using quadratic models, while a linear model effectively predicted the whiteness index (WI). Other color parameters, however, could not be reliably modeled. Over a 12‐month storage period, RS‐treated samples exhibited superior color stability compared to those processed by CA. Furthermore, an adaptive neuro‐fuzzy inference system (ANFIS) model was employed to predict thiobarbituric acid (TBA) values based on L*, a*, and b* parameters. Among the tested models, a reduced sixth‐order model provided the most accurate TBA predictions with minimal error. Future research may explore the use of artificial neural networks and deep learning approaches for identifying color components in dairy products to evaluate their quality characteristics.

## Author Contributions

**Atheer A. A. Al‐Mtury:** methodology, formal analysis, data curation, investigation, writing – original draft, writing – review and editing. **Asaad R. Al‐Hilphy:** methodology, formal analysis, data curation, investigation, visualization. **Ammar B. Altemimi:** methodology, conceptualization, supervision, data curation, formal analysis, writing – original draft, writing – review and editing. **Zheng Ruan:** methodology, formal analysis, writing – original draft, writing – review and editing. **Sabah M. Al‐Shatty:** conceptualization, formal analysis, data curation. **Mohammad Ali Hesarinejad:** methodology, software, writing – review and editing. **Tarek Gamal Abedelmaksoud:** methodology, software, conceptualization, data curation, writing – original draft, writing – review and editing.

## Conflicts of Interest

The authors declare no conflicts of interest.

## Supporting information


Tables S1–S10.

## Data Availability

Data will be made available on request.

## References

[fsn370768-bib-0001] Abbas, M. Z. , I. A. Sajjad , B. Hussain , et al. 2022. “An Adaptive‐Neuro Fuzzy Inference System Based‐Hybrid Technique for Performing Load Disaggregation for Residential Customers.” Scientific Reports 12, no. 1: 2384. 10.1038/s41598-022-06381-7. 35149746 PMC8837745

[fsn370768-bib-0002] Abdulla, M. Z. , L. C. Guan , K. C. Lim , and A. A. Karim . 2004. “The Application of Computer Vision and Tomographic Radar Imaging for Assessing Physical Properties of Food.” Journal of Food Engineering 61: 125–135. 10.1016/S0260-8774(03)00194-8.

[fsn370768-bib-0003] Al‐Hilphy, A. R. , H. I. Ali , S. A. Al‐IEssa , J. M. Lorenzo , F. J. Barba , and M. Gavahian . 2021. “Refractance Window (RW) Concentration of Milk‐Part II: Computer Vision Approach for Optimizing Microbial and Sensory Qualities.” Journal of Food Processing and Preservation 45, no. 9: e15702. 10.1111/jfpp.15702.

[fsn370768-bib-0004] Arunachalam, V. , D. C. Salgaonkar , N. V. Kevat , B. V. Walawalkar , and B. Das . 2022. “Quantification of Betacyanin Content Variation of Amaranth Varieties by an Android App, Colorimeter, and Infrared Spectroscopy.” Chinese Journal of Analytical Chemistry 50, no. 10: 100145. 10.1016/j.cjac.2022.100145.

[fsn370768-bib-0005] Costa, C. , F. Antonucci , F. Pallottino , J. Aguzzi , D. Sun , and P. Menesatti . 2011. “Shape Analysis of Agricultural Products: A Review of Recent Research Advances and Potential Application to Computer Vision.” Food and Bioprocess Technology 4, no. 5: 673–692. 10.1007/s11947-011-0556-0.

[fsn370768-bib-0006] Cruz, R. , V. Pereira , T. Pinho , I. M. P. L. V. O. Ferreira , C. Novais , and S. Casal . 2022. “Safety and Quality of Canned Sardines After Opening: A Shelf‐Stability Study.” Food 11, no. 7: 991. 10.3390/foods11070991. PMC8997536 35407078

[fsn370768-bib-0007] Du, C. J. , and D. W. Sun . 2004. “Recent Developments in the Applications of Image Processing Techniques for Food Quality Evaluation.” Trends in Food Science and Technology 15, no. 5: 230–249. 10.1016/j.tifs.2003.10.006.

[fsn370768-bib-0008] Galhoum, G. F. 2020. “Production of Canned Tuna Using Local Tuna Species.” Food Science & Nutrition Technology 5, no. 2: 14p. 10.23880/fsnt.

[fsn370768-bib-0009] Gómez‐Limia, L. , J. Carballo , M. Rodríguez‐González , and S. Martínez . 2022. “Impact of the Filling Medium on the Colour and Sensory Characteristics of Canned European Eels (*Anguilla* L.).” Food 11, no. 8: 1–19. 10.3390/foods11081115. PMC9027171 35454703

[fsn370768-bib-0010] Gulrajani, M. L. 2010. Colour Measurement Principles Advances and Industrial Applications, 402. Woodhead Publishing Limited.

[fsn370768-bib-0011] Hatcher, D. W. , S. J. Symons , and U. Manivannan . 2004. “Developments in the Use of Image Analysis for the Assessment of Oriental Noodle Appearance and Color.” Journal of Food Engineering 61: 109–117. 10.1016/S0260-8774(03)00192-4.

[fsn370768-bib-0024] Khuri, A.I. , and J. A. Cornell . 2018. “Response Surfaces: Designs and Analyses, Routledge.” 10.1201/9780203740774.

[fsn370768-bib-0012] Martinez, B. , M. Trigo , A. Rodrigous , and S. P. Aubourg . 2024. “Influence of Cuttlefish‐Ink Extract on Canned Golden Sea Bream ( *Sparus aurata* ) Quality.” Food 13: 1685. 10.3390/foods13111685. PMC11171682 38890914

[fsn370768-bib-0013] Miao, Y. , G. Hu , H. Huang , Y. Li , and Y. Fu . 2023. “Effects of Tea Polyphenols Combined With Thermosonication on the Population of *Salmonella enterica* in Fresh‐Cut Wax Gourd During Storage and Its ANFIS Survival Model.” Applied Sciences 13, no. 8: 5087. 10.3390/app13085087.

[fsn370768-bib-0014] Morsy, M. K. 2016. “Quality Enhancement of Canned Little Tunny Fish (*Euthynnus alletteratus*) by Whitening Solutions, Pre‐Cooking Time and Filling Medium.” Journal of Food Processing and Technology 7, no. 11: 1–8. 10.4172/2157-7110.1000632.

[fsn370768-bib-0015] Nosratabadi, S. , S. Ardabili , Z. Lakner , C. Mako , and A. Mosavi . 2021. “Prediction of Food Production Using Machine Learning Algorithms of Multilayer Perceptron and ANFIS.” Agriculture 11, no. 5: 408. 10.3390/agriculture11050408.

[fsn370768-bib-0016] Pathare, P. B. , U. L. Opara , and F. A. Al‐Said . 2013. “Color Measurement and Analysis in Fresh and Processed Foods: A Review.” Food and Bioprocess Technology 6, no. 1: 36–60. 10.1007/s11947-012-0867-9.

[fsn370768-bib-0017] Pedreschi, F. , J. M. Aguilera , and C. A. Brown . 2000. “Characterization of Food Surfaces Using Scale‐Sensitive Fractal Analysis.” Journal of Food Process Engineering 23, no. 2: 127–143. 10.1111/J.1745-4530.2000.TB00507.X.

[fsn370768-bib-0018] Saad, M. S. , I. S. Islam , and I. A. Ibrahim . 2021. “Chemical Evaluation of Imported Fish.” Benha Veterinary Medical Journal 40, no. 2: 53–55. 10.21608/BVMJ.2021.68335.1379.

[fsn370768-bib-0019] Sahin, S. , and S. G. Sumnu . 2006. Physical Properties of Foods, 257. Springer Science+Business Media, LLC. 10.1007/0-387-30808-3.

[fsn370768-bib-0020] Shihabudheen, K. V. , and G. N. Pillai . 2018. “Recent Advances in Neuro‐Fuzzy System: A Survey.” Knowledge‐Based Systems 152: 136–162. 10.1016/j.knosys.2018.04.014.

[fsn370768-bib-0022] Spence, C. 2015. “On the Psychological Impact of Food Colour.” Flavour 4: 21–47. 10.1186/s13411-015-0031-3.

[fsn370768-bib-0023] Villamarín, E. , B. Martínez , M. Trigo , and S. P. Aubourg . 2023. “Influence of Different Previous Frozen Holding Periods on the Canned Fish Quality.” Food 12: 4117. 10.3390/foods12224117. PMC10670115 38002175

