# Supplementary material for: Optimization of Canned Talang Queenfish Color Sterilized by Rotary Retort: Storage Stability, Artificial Intelligence—Adaptive Neuro Fuzzy Inference Systems Modeling TBA Based on Color Attributes
Source: Food Sci Nutr. 2025 Aug 17;13(8):e70768. doi: 10.1002/fsn3.70768 (PMC12358691; doi:10.1002/fsn3.70768)
Supplement: Supplementary file 1 — Tables S1–S10. [file FSN3-13-e70768-s001.docx]

**Supplementary**

Table S1. Analysis variance (ANOVA) and significant coefficient for L value

| **Source** | **Sum of Squares** | **df** | **Mean Square** | **F-value** | **p-value** |  |
| --- | --- | --- | --- | --- | --- | --- |
| **Model** | 15.12 | 5 | 3.02 | 11.64 | 0.0028 | significant |
| A-Temperature | 13.50 | 1 | 13.50 | 51.97 | 0.0002 |  |
| B-Time | 0.0150 | 1 | 0.0150 | 0.0577 | 0.8170 |  |
| AB | 0.0100 | 1 | 0.0100 | 0.0385 | 0.8500 |  |
| A² | 0.2317 | 1 | 0.2317 | 0.8921 | 0.3764 |  |
| B² | 0.8043 | 1 | 0.8043 | 3.10 | 0.1219 |  |
| **Residual** | 1.82 | 7 | 0.2598 |  |  |  |
| Lack of Fit | 0.2783 | 3 | 0.0928 | 0.2409 | 0.8641 | not significant |
| Pure Error | 1.54 | 4 | 0.3850 |  |  |  |
| **Cor Total** | 16.94 | 12 |  |  |  |  |
| **R²** | 0.8927 |  |  |  |  |  |
| **Adjusted R²** | 0.8160 |  |  |  |  |  |
| **Predicted R²** | 0.7493 |  |  |  |  |  |
| **Adeq Precision** | 10.2229 |  |  |  |  |  |
| **Std. Dev.** | 0.5097 |  |  |  |  |  |
| **Mean** | 54.40 |  |  |  |  |  |
| **C.V. %** | 0.9369 |  |  |  |  |  |

Table S2. Analysis variance (ANOVA) and significant coefficient for a value

| **Source** | **Sum of Squares** | **df** | **Mean Square** | **F-value** | **p-value** |  |
| --- | --- | --- | --- | --- | --- | --- |
| **Model** | 0.0550 | 5 | 0.0110 | 1.24 | 0.3824 | not significant |
| A-Temperature | 0.0067 | 1 | 0.0067 | 0.7532 | 0.4142 |  |
| B-Time | 1.388E-17 | 1 | 1.388E-17 | 1.568E-15 | 1.0000 |  |
| AB | 0.0100 | 1 | 0.0100 | 1.13 | 0.3231 |  |
| A² | 0.0119 | 1 | 0.0119 | 1.34 | 0.2851 |  |
| B² | 0.0119 | 1 | 0.0119 | 1.34 | 0.2851 |  |
| **Residual** | 0.0620 | 7 | 0.0089 |  |  |  |
| Lack of Fit | 0.0140 | 3 | 0.0047 | 0.3876 | 0.7689 | not significant |
| Pure Error | 0.0480 | 4 | 0.0120 |  |  |  |
| **Cor Total** | 0.1169 | 12 |  |  |  |  |
| **R²** | 0.4701 |  |  |  |  |  |
| **Adjusted R²** | 0.0917 |  |  |  |  |  |
| **Predicted R²** | -0.7966 |  |  |  |  |  |
| **Adeq Precision** | 3.3541 |  |  |  |  |  |
| **Std. Dev.** | 0.0941 |  |  |  |  |  |
| **Mean** | 1.52 |  |  |  |  |  |
| **C.V. %** | 6.21 |  |  |  |  |  |

Table S3. Analysis variance (ANOVA) and significant coefficient for b value

| **Source** | **Sum of Squares** | **df** | **Mean Square** | **F-value** | **p-value** |  |
| --- | --- | --- | --- | --- | --- | --- |
| **Model** | 0.8929 | 5 | 0.1786 | 1.96 | 0.2015 | not significant |
| A-Temperature | 0.4267 | 1 | 0.4267 | 4.69 | 0.0670 |  |
| B-Time | 0.1667 | 1 | 0.1667 | 1.83 | 0.2178 |  |
| AB | 0.0400 | 1 | 0.0400 | 0.4400 | 0.5283 |  |
| A² | 0.1303 | 1 | 0.1303 | 1.43 | 0.2701 |  |
| B² | 0.2208 | 1 | 0.2208 | 2.43 | 0.1631 |  |
| **Residual** | 0.6363 | 7 | 0.0909 |  |  |  |
| Lack of Fit | 0.0283 | 3 | 0.0094 | 0.0621 | 0.9772 | not significant |
| Pure Error | 0.6080 | 4 | 0.1520 |  |  |  |
| **Cor Total** | 1.53 | 12 |  |  |  |  |
| **R²** | 0.5839 |  |  |  |  |  |
| **Adjusted R²** | 0.2867 |  |  |  |  |  |
| **Predicted R²** | 0.2566 |  |  |  |  |  |
| **Adeq Precision** | 4.5566 |  |  |  |  |  |
| **Std. Dev.** | 0.3015 |  |  |  |  |  |
| **Mean** | 18.19 |  |  |  |  |  |
| **C.V. %** | 1.66 |  |  |  |  |  |

Table S4. Analysis variance (ANOVA) and significant coefficient for ΔE

| **Source** | **Sum of Squares** | **df** | **Mean Square** | **F-value** | **p-value** |  |
| --- | --- | --- | --- | --- | --- | --- |
| **Model** | 9.80 | 5 | 1.96 | 9.31 | 0.0053 | significant |
| A-Temperature | 8.52 | 1 | 8.52 | 40.45 | 0.0004 |  |
| B-Time | 0.0025 | 1 | 0.0025 | 0.0120 | 0.9159 |  |
| AB | 0.0202 | 1 | 0.0202 | 0.0958 | 0.7659 |  |
| A² | 0.0511 | 1 | 0.0511 | 0.2425 | 0.6375 |  |
| B² | 0.8659 | 1 | 0.8659 | 4.11 | 0.0822 |  |
| **Residual** | 1.47 | 7 | 0.2105 |  |  |  |
| Lack of Fit | 0.2740 | 3 | 0.0913 | 0.3045 | 0.8219 | not significant |
| Pure Error | 1.20 | 4 | 0.3000 |  |  |  |
| **Cor Total** | 11.27 | 12 |  |  |  |  |
| **R²** | 0.8693 |  |  |  |  |  |
| **Adjusted R²** | 0.7759 |  |  |  |  |  |
| **Predicted R²** | 0.6664 |  |  |  |  |  |
| **Adeq Precision** | 9.7339 |  |  |  |  |  |
| **Std. Dev.** | 0.4589 |  |  |  |  |  |
| **Mean** | 29.33 |  |  |  |  |  |
| **C.V. %** | 1.56 |  |  |  |  |  |

Table S5. Analysis variance (ANOVA) and significant coefficient for C value

| **Source** | **Sum of Squares** | **df** | **Mean Square** | **F-value** | **p-value** |  |
| --- | --- | --- | --- | --- | --- | --- |
| **Model** | 0.8896 | 5 | 0.1779 | 1.99 | 0.1977 | not significant |
| A-Temperature | 0.4327 | 1 | 0.4327 | 4.83 | 0.0639 |  |
| B-Time | 0.1655 | 1 | 0.1655 | 1.85 | 0.2161 |  |
| AB | 0.0366 | 1 | 0.0366 | 0.4093 | 0.5427 |  |
| A² | 0.1361 | 1 | 0.1361 | 1.52 | 0.2574 |  |
| B² | 0.2107 | 1 | 0.2107 | 2.35 | 0.1689 |  |
| **Residual** | 0.6266 | 7 | 0.0895 |  |  |  |
| Lack of Fit | 0.0299 | 3 | 0.0100 | 0.0667 | 0.9747 | not significant |
| Pure Error | 0.5968 | 4 | 0.1492 |  |  |  |
| **Cor Total** | 1.52 | 12 |  |  |  |  |
| **R²** | 0.5867 |  |  |  |  |  |
| **Adjusted R²** | 0.2915 |  |  |  |  |  |
| **Predicted R²** | 0.2517 |  |  |  |  |  |
| **Adeq Precision** | 4.5890 |  |  |  |  |  |
| **Std. Dev.** | 0.2992 |  |  |  |  |  |
| **Mean** | 18.26 |  |  |  |  |  |
| **C.V. %** | 1.64 |  |  |  |  |  |

Table S6. Analysis variance (ANOVA) and significant coefficient for h value

| **Source** | **Sum of Squares** | **df** | **Mean Square** | **F-value** | **p-value** |  |
| --- | --- | --- | --- | --- | --- | --- |
| **Model** | 0.5759 | 5 | 0.1152 | 1.14 | 0.4219 | not significant |
| A-Temperature | 0.0080 | 1 | 0.0080 | 0.0791 | 0.7867 |  |
| B-Time | 0.0113 | 1 | 0.0113 | 0.1115 | 0.7482 |  |
| AB | 0.1329 | 1 | 0.1329 | 1.31 | 0.2895 |  |
| A² | 0.0631 | 1 | 0.0631 | 0.6229 | 0.4559 |  |
| B² | 0.2112 | 1 | 0.2112 | 2.09 | 0.1918 |  |
| **Residual** | 0.7085 | 7 | 0.1012 |  |  |  |
| Lack of Fit | 0.1262 | 3 | 0.0421 | 0.2889 | 0.8322 | not significant |
| Pure Error | 0.5823 | 4 | 0.1456 |  |  |  |
| **Cor Total** | 1.28 | 12 |  |  |  |  |
| **R²** | 0.4484 |  |  |  |  |  |
| **Adjusted R²** | 0.0543 |  |  |  |  |  |
| **Predicted R²** | -0.6538 |  |  |  |  |  |
| **Adeq Precision** | 3.1915 |  |  |  |  |  |
| **Std. Dev.** | 0.3181 |  |  |  |  |  |
| **Mean** | 4.77 |  |  |  |  |  |
| **C.V. %** | 6.68 |  |  |  |  |  |

Table S7. Analysis variance (ANOVA) and significant coefficient for WI

| **Source** | **Sum of Squares** | **df** | **Mean Square** | **F-value** | **p-value** |  |
| --- | --- | --- | --- | --- | --- | --- |
| **Model** | 10.07 | 2 | 5.03 | 15.17 | 0.0009 | significant |
| A-Temperature | 10.07 | 1 | 10.07 | 30.33 | 0.0003 |  |
| B-Time | 0.0013 | 1 | 0.0013 | 0.0038 | 0.9523 |  |
| **Residual** | 3.32 | 10 | 0.3319 |  |  |  |
| Lack of Fit | 1.90 | 6 | 0.3165 | 0.8916 | 0.5723 | not significant |
| Pure Error | 1.42 | 4 | 0.3550 |  |  |  |
| **Cor Total** | 13.39 | 12 |  |  |  |  |
| **R²** | 0.7521 |  |  |  |  |  |
| **Adjusted R²** | 0.7025 |  |  |  |  |  |
| **Predicted R²** | 0.5459 |  |  |  |  |  |
| **Adeq Precision** | 9.4651 |  |  |  |  |  |
| **Std. Dev.** | 0.5761 |  |  |  |  |  |
| **Mean** | 50.88 |  |  |  |  |  |
| **C.V. %** | 1.13 |  |  |  |  |  |

Table S8. Analysis variance (ANOVA) and significant coefficient for YI

| **Source** | **Sum of Squares** | **df** | **Mean Square** | **F-value** | **p-value** |  |
| --- | --- | --- | --- | --- | --- | --- |
| **Model** | 7.91 | 5 | 1.58 | 1.75 | 0.2408 | not significant |
| A-Temperature | 2.34 | 1 | 2.34 | 2.59 | 0.1515 |  |
| B-Time | 0.9760 | 1 | 0.9760 | 1.08 | 0.3327 |  |
| AB | 0.4233 | 1 | 0.4233 | 0.4697 | 0.5152 |  |
| A² | 0.2432 | 1 | 0.2432 | 0.2698 | 0.6195 |  |
| B² | 4.08 | 1 | 4.08 | 4.53 | 0.0709 |  |
| **Residual** | 6.31 | 7 | 0.9013 |  |  |  |
| Lack of Fit | 0.8092 | 3 | 0.2697 | 0.1962 | 0.8940 | not significant |
| Pure Error | 5.50 | 4 | 1.38 |  |  |  |
| **Cor Total** | 14.22 | 12 |  |  |  |  |
| **R²** | 0.5562 |  |  |  |  |  |
| **Adjusted R²** | 0.2391 |  |  |  |  |  |
| **Predicted R²** | -0.0173 |  |  |  |  |  |
| **Adeq Precision** | 4.9492 |  |  |  |  |  |
| **Std. Dev.** | 0.9494 |  |  |  |  |  |
| **Mean** | 47.79 |  |  |  |  |  |
| **C.V. %** | 1.99 |  |  |  |  |  |

Table S9. Analysis variance (ANOVA) and significant coefficient for BI

| **Source** | **Sum of Squares** | **df** | **Mean Square** | **F-value** | **p-value** |  |
| --- | --- | --- | --- | --- | --- | --- |
| **Model** | 7.33 | 5 | 1.47 | 1.74 | 0.2439 | not significant |
| A-Temperature | 2.44 | 1 | 2.44 | 2.90 | 0.1326 |  |
| B-Time | 0.9804 | 1 | 0.9804 | 1.16 | 0.3165 |  |
| AB | 0.2729 | 1 | 0.2729 | 0.3238 | 0.5871 |  |
| A² | 0.3906 | 1 | 0.3906 | 0.4635 | 0.5179 |  |
| B² | 3.62 | 1 | 3.62 | 4.30 | 0.0768 |  |
| **Residual** | 5.90 | 7 | 0.8428 |  |  |  |
| Lack of Fit | 1.02 | 3 | 0.3394 | 0.2781 | 0.8394 | not significant |
| Pure Error | 4.88 | 4 | 1.22 |  |  |  |
| **Cor Total** | 13.23 | 12 |  |  |  |  |
| **R²** | 0.5541 |  |  |  |  |  |
| **Adjusted R²** | 0.2356 |  |  |  |  |  |
| **Predicted R²** | -0.1619 |  |  |  |  |  |
| **Adeq Precision** | 4.9491 |  |  |  |  |  |
| **Std. Dev.** | 0.9181 |  |  |  |  |  |
| **Mean** | 41.33 |  |  |  |  |  |
| **C.V. %** | 2.22 |  |  |  |  |  |

Table S10. ANOVA analysis of the impact color attributes and reduced sixth model on the TBA.

| **Source** | **Sum of Squares** | **df** | **Mean Square** | **F-value** | **p-value** |  |
| --- | --- | --- | --- | --- | --- | --- |
| **Model** | 0.2495 | 37 | 0.0067 | 7153.14 | < 0.0001 | significant |
| A-L* | 0.0005 | 1 | 0.0005 | 556.41 | < 0.0001 |  |
| B-a* | 0.0083 | 1 | 0.0083 | 8763.04 | < 0.0001 |  |
| C-b* | 3.948E-06 | 1 | 3.948E-06 | 4.19 | 0.0450 |  |
| AB | 0.0010 | 1 | 0.0010 | 1064.30 | < 0.0001 |  |
| A² | 0.0088 | 1 | 0.0088 | 9343.36 | < 0.0001 |  |
| B² | 0.0023 | 1 | 0.0023 | 2470.26 | < 0.0001 |  |
| A²BC | 0.0000 | 1 | 0.0000 | 26.13 | < 0.0001 |  |
| A³B | 0.0000 | 1 | 0.0000 | 13.83 | 0.0004 |  |
| A³C | 0.0000 | 1 | 0.0000 | 14.08 | 0.0004 |  |
| AB³ | 0.0000 | 1 | 0.0000 | 34.56 | < 0.0001 |  |
| BC³ | 0.0001 | 1 | 0.0001 | 53.86 | < 0.0001 |  |
| A²BC² | 0.0000 | 1 | 0.0000 | 12.07 | 0.0009 |  |
| AB²C² | 9.766E-06 | 1 | 9.766E-06 | 10.36 | 0.0021 |  |
| A³B² | 0.0000 | 1 | 0.0000 | 11.94 | 0.0010 |  |
| A³C² | 0.0000 | 1 | 0.0000 | 12.36 | 0.0008 |  |
| A²C³ | 0.0000 | 1 | 0.0000 | 13.74 | 0.0004 |  |
| B³C² | 0.0000 | 1 | 0.0000 | 16.04 | 0.0002 |  |
| A⁴C | 9.993E-06 | 1 | 9.993E-06 | 10.60 | 0.0018 |  |
| AB⁴ | 0.0000 | 1 | 0.0000 | 27.02 | < 0.0001 |  |
| B⁵ | 0.0000 | 1 | 0.0000 | 22.55 | < 0.0001 |  |
| A³B³ | 0.0000 | 1 | 0.0000 | 26.24 | < 0.0001 |  |
| A³BC² | 0.0000 | 1 | 0.0000 | 28.95 | < 0.0001 |  |
| A²BC³ | 0.0000 | 1 | 0.0000 | 20.23 | < 0.0001 |  |
| AB³C² | 0.0000 | 1 | 0.0000 | 10.77 | 0.0017 |  |
| AB²C³ | 0.0000 | 1 | 0.0000 | 42.61 | < 0.0001 |  |
| A⁴B² | 5.932E-06 | 1 | 5.932E-06 | 6.29 | 0.0148 |  |
| A⁴BC | 0.0000 | 1 | 0.0000 | 15.23 | 0.0002 |  |
| A⁴C² | 0.0000 | 1 | 0.0000 | 15.46 | 0.0002 |  |
| A²B⁴ | 0.0000 | 1 | 0.0000 | 15.50 | 0.0002 |  |
| A²C⁴ | 9.894E-06 | 1 | 9.894E-06 | 10.49 | 0.0019 |  |
| ABC⁴ | 0.0001 | 1 | 0.0001 | 59.14 | < 0.0001 |  |
| A⁵B | 0.0000 | 1 | 0.0000 | 23.48 | < 0.0001 |  |
| A⁵C | 0.0000 | 1 | 0.0000 | 25.93 | < 0.0001 |  |
| B⁵C | 0.0000 | 1 | 0.0000 | 21.98 | < 0.0001 |  |
| BC⁵ | 0.0000 | 1 | 0.0000 | 17.01 | 0.0001 |  |
| A⁶ | 0.0000 | 1 | 0.0000 | 38.92 | < 0.0001 |  |
| B⁶ | 0.0000 | 1 | 0.0000 | 31.93 | < 0.0001 |  |
| **Residual** | 0.0001 | 62 | 9.428E-07 |  |  |  |
| **Cor Total** | 0.2496 | 99 |  |  |  |  |
| **Mean** | 0.2147 |  |  |  |  |  |
| **C.V. %** | 0.4522 |  |  |  |  |  |
| **Adjusted R²** | 0.9996 |  |  |  |  |  |
| **Predicted R²** | 0.9925 |  |  |  |  |  |
| **Adeq Precision** | 449.0511 |  |  |  |  |  |
